# Hemodynamic Effect of Volatile Sedation in Acute Respiratory Distress Syndrome Patients Undergoing Venovenous Extracorporeal Membrane Oxygenation: A Pilot Observational Study

**DOI:** 10.1097/ALN.0000000000005680

**Published:** 2025-10-14

**Authors:** Marco Giani, Benedetta Fumagalli, Elisa Zoe Battistelli, Marta Frazzei, Giacomo Grasselli, Matteo Pozzi, Emanuele Rezoagli, Giuseppe Foti

**Affiliations:** 1 University of Milan-Bicocca, Milan, Italy; 2 Fondazione IRCCS (Scientific Institute for Research, Hospitalization and Healthcare) San Gerardo dei Tintori, Monza, Italy; 3 Fondazione IRCCS (Scientific Institute for Research, Hospitalization and Healthcare) Ca’ Granda Ospedale Maggiore Policlinico, Milan, Italy; 4 University of Milan, Milan, Italy

## To the Editor:

Achieving an adequate sedation plan for patients with acute respiratory distress syndrome (ARDS) requiring venovenous extracorporeal membrane oxygenation (ECMO) is often challenging. Intravenous (IV) agents like propofol and midazolam are commonly used, but they are associated with well-known adverse effects, including tachyphylaxis, myotoxicity, and accumulation.^1,2^ Moreover, specific considerations apply to IV drugs administered to patients on ECMO. Several studies have shown an increased requirement for lipophilic sedatives during ECMO support, attributable to sequestration within the circuit and to increased volume of distribution.^3^ Inhaled sedation has emerged as a feasible alternative to IV sedation in patients with respiratory failure requiring venovenous ECMO and prolonged controlled mechanical ventilation.^4,5^

Volatile anesthetics offer some advantages, including rapid onset and offset, predominant pulmonary elimination with minimal hepatic metabolism, and absence of active metabolites, resulting in shorter awakening and extubation times.^6,7^ Furthermore, volatile agents are associated with a lower risk of delirium and dependence compared with benzodiazepines.^8^

The hemodynamic impact of the transition from IV to volatile sedation in ARDS patients on ECMO support is unknown.

For this reason, we conducted a prospective observational study including adult ARDS patients receiving volatile sedation with isoflurane during venovenous ECMO at the general intensive care unit (ICU) of IRCCS (*i.e.*, Scientific Institute for Research, Hospitalization and Healthcare) San Gerardo dei Tintori (Monza, Italy), between May 2022 and February 2025. The aim of the study was to evaluate hemodynamic outcomes during the transition from IV to volatile sedation in patients undergoing venovenous ECMO. The primary outcome was systolic arterial pressure. Secondary outcomes included other hemodynamic parameters (heart rate, pulmonary artery pressure, pulmonary capillary wedge pressure, cardiac output, and vasopressor requirements) as well as sedation adequacy (assessed by Bispectral Index [BIS]), gas exchange, and the occurrence of gas-related adverse effects. The study was approved by the local ethics committee (May 2022, reference 3689).

Hemodynamic data were prospectively collected every 2 h as part of routine ECMO monitoring. Analysis was restricted to the 24 h preceding and following AnaConDa (Sedana Medical, Sweden) connection. Vasopressor management followed standard clinical practice, without a formalized weaning protocol, and targeted a systolic arterial pressure higher than 85 and mean arterial pressure higher than 60 mmHg. Only data from patients who were switched to volatile sedation were included in the analysis. Patients were excluded if they were pregnant, had a known or suspected predisposition to malignant hyperthermia, had severe hepatic failure, and were not receiving controlled mechanical ventilation before isoflurane initiation.

Isoflurane was administered using an anesthetic conserving device (AnaConDa). Sedation depth was assessed using the BIS (target 35 to 50). Comprehensive information on volatile sedation, including delivery method and clinical use at our institution, is provided in the Supplemental Digital Content methods (https://links.lww.com/ALN/E153), along with details on data collection.

To evaluate within-subject differences in hemodynamic parameters before and after isoflurane initiation, we used a linear mixed-effects model, specifying sedation type (IV *vs*. volatile) as a fixed effect and patient as a random effect. Due to the exploratory nature of this pilot study and the limited number of patients, no formal correction for multiple testing was applied. Analyses were conducted using JMP Pro 18 (SAS, USA).

Seventeen of 46 consecutive venovenous ECMO patients met the inclusion criteria and were included in the analysis (Supplemental Digital Content fig. S1, https://links.lww.com/ALN/E153). The median age was 51 yr, and 18% of patients were women. Demographic details, disease etiology, and duration of IV and volatile sedation are provided in the Supplemental Digital Content results (table S1, https://links.lww.com/ALN/E153).

Doses of sedatives, opioids, and neuromuscular blocking agents are summarized in table 1. The target level of sedation was achieved in all patients during isoflurane administration, with a median end-tidal concentration of 0.9% [0.8 to 1.0%]; however, three patients (18%) required a concurrent midazolam infusion. BIS values did not differ before and after switching to isoflurane (39 [35 to 44] and 42 [38 to 46], respectively; *P* = 0.09).

**Table 1.** Dosages of Sedative Agents, Neuromuscular Blocking Agents, Opioids, Vasopressors, and Inotropes

	Intravenous Sedation	Volatile Sedation
Sedative agents		
Isoflurane		
No. (%)	—	17 (100%)
Infusion rate, ml/h	—	15 [13–18]
End tidal, %	—	0.9 [0.8–1]
Propofol		
No. (%)	17 (100%)	—
Infusion rate, µg · kg^−1^ · min^−1^	47 [43–58]	—
Midazolam		
No. (%)	7 (41)	3 (18)
Infusion rate, mg/h	4 [3–5]	3 [1–4]*
Opioids		
Fentanyl		
No. (%)	17 (100%)	17 (100%)
Infusion rate, µg/h	100 [100–100]	100 [100–100]
Neuromuscular blocking agents		
Rocuronium		
No. (%)	8 (47%)	8 (47%)
Infusion rate, mg/h	30 [30–40]	30 [30–40]
Cisatracurium		
No. (%)	9 (53%)	9 (53%)
Infusion rate, mg/h	10 [8–10]	10 [8–10]
Vasopressors and inotropes		
Norepinephrine		
No. (%)	13 (76)	9 (53)
Infusion rate, µg · kg^−1^ · min^−1^	0.11 [0.07–0.16]	0.07 [0.04–0.10]*
Dobutamine		
No. (%)	2 (12)	1 (6)
Infusion rate, µg · kg^−1^ · min^−1^	8.0 [7.0–8.5]	8.0 [7.3–8.0]
Hemodynamic parameters		
Cardiac output, l/min	6.7 [5.5–8.2]	6.9 [6.1–8.3]
Heart rate, beats/min	86 [73–95]	92 [83–104]*
Systolic arterial pressure, mmHg	114 [102–124]	113 [101–124]
Mean pulmonary arterial pressure, mmHg	26 [24–29]	27 [23–31]
Wedge pressure, mmHg	12 [11–15]	11 [8–13]

Data are expressed as median [interquartile range] and absolute (relative) frequency.

* *P* < 0.05 *vs.* intravenous sedation.

**Fig. 1.**
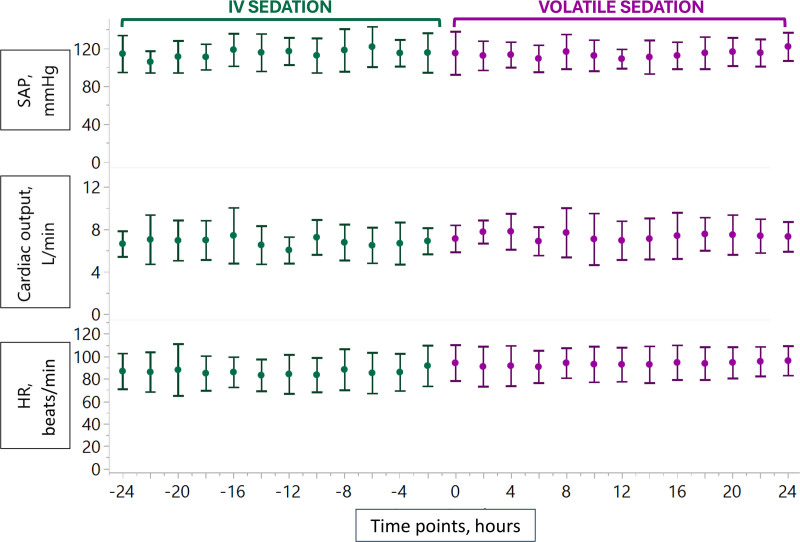
Hemodynamic parameters during the last 24 h of intravenous (IV) sedation and the first 24 h of volatile sedation. HR, heart rate; SAP, systolic arterial pressure.

Table 1 also reports hemodynamic parameters and vasopressors requirements. Noradrenaline doses significantly decreased during the volatile phase (0.07 µg · kg^−1^ · min^−1^ *vs*. 0.11 µg · kg^−1^ · min^−1^; mean difference, 0.04; 95% CI, 0.03 to 0.05; *P* < 0.001). No significant changes were observed in systolic arterial pressure (mean difference, 2; 95% CI, −2 to 5; *P* = 0.31), pulmonary mean arterial pressure (−0.4; 95% CI, −1.2 to 0.4; *P* = 0.321), pulmonary capillary wedge pressure (0.2; 95% CI, −1.2 to 1.6; *P* = 0.747), or cardiac output (−0.03; 95% CI, –0.3 to 0.2; *P* = 0.828). A mild but statistically significant increase in heart rate was noted following the transition to isoflurane (mean difference, −6 beats/min; 95% CI, −7 to −4; *P* < 0.001). Trends in heart rate, systolic arterial pressure, and cardiac output before and after the transition to volatile sedation are shown in figure 1. All patients received ultraprotective ventilation throughout both sedation phases. Additional details regarding ventilatory settings, ECMO parameters, and gas exchange are provided in Supplemental Digital Content table S2 (https://links.lww.com/ALN/E153). Isoflurane administration was not associated with any adverse events.

In this cohort of ARDS patients requiring venovenous ECMO support, we evaluated the hemodynamic effects of transitioning from IV to inhaled sedation. An adequate sedation level was achieved during both phases, and no adverse events related to isoflurane were observed. The switch in sedation regimen did not appear to significantly impact systolic arterial pressure or cardiac output, while a modest increase in heart rate was recorded. Notably, while similar arterial pressure targets were maintained during the two study phases, volatile sedation showed a significant trend toward lower overall vasopressor requirements than IV sedation.

Our findings are consistent with those reported in other studies. A prospective crossover study comparing IV to volatile sedation in intubated ICU patients found an overall comparable hemodynamic profile.^9^ Similarly, a retrospective study from our research group demonstrated the feasibility of isoflurane sedation in ARDS patients on venovenous ECMO receiving ultraprotective ventilation with stable hemodynamic parameters.^4^

In contrast, the recently published SESAR Sevoflurane for Sedation in ARDS (SESAR) trial,^10^ investigating volatile sedation with sevoflurane in non-ECMO ARDS patients, reported increased norepinephrine requirements and higher incidence of acute kidney injury, potentially contributing to the greater 90-day mortality observed in the sevoflurane group. However, the SESAR trial did not include systematic monitoring of sedation depth using objective parameters such as BIS or end-tidal sevoflurane concentrations. This may have influenced the study findings, given the potentially unpredictable pharmacokinetics of volatile agents when delivered using devices such as AnaConDa. It should be noted that AnaConDa delivery of isoflurane in ECMO patients undergoing protective ventilation likely exhibits markedly different pharmacokinetics than sevoflurane delivered *via* anesthesia machines to spontaneously breathing ICU patients. Unlike anesthesia machines, volatile agent concentration with AnaConDa is inversely related to patient ventilation, potentially resulting in variable drug delivery. Therefore, oversedation by sevoflurane, potentially leading to increased use of vasopressors, as observed in the SESAR trial, cannot be ruled out. For similar reasons, the use of volatile agents in the presence of a variable minute ventilation, such as in spontaneously breathing ICU patients, is debatable. These considerations underscore the necessity for specialized training in the management of volatile sedation, comparable with the one received during anesthesiology training.

The observational design of our study, the small sample size, and the sequential nature of the interventions limit causal inference. Moreover, due to the statistical methodology employed, the influence of confounders, such as baseline patient characteristics, changes in illness severity over time, and depth of anesthesia, on hemodynamic trends cannot be excluded. Selection bias could also represent a potential confounder, although its impact is partially mitigated by the repeated-measures design, in which each patient serves as their own control. Nevertheless, the small sample size and single-center setting remain important limitations. Despite these limitations, our findings suggest that, in patients requiring prolonged deep sedation and thus at risk of complications from extended IV drug use, volatile sedation, when administered under controlled conditions at experienced centers, may represent a valuable and safe alternative without adverse effects on hemodynamics. Larger multicenter trials with standardized sedation and vasopressor protocols, extended monitoring, and clinically meaningful endpoints (*e.g.*, ventilator-free days, delirium incidence, ICU length of stay, and mortality) are needed to confirm and generalize our observations.

## Research Support

Prof. Grasselli reports receiving payments for lectures from Getinge (Göteborg, Sweden), Draeger (Lübeck, Germany), and Fisher & Paykel (Auckland, New Zealand) as well as unrestricted research grants from Fisher & Paykel, all outside the scope of the current work.

## Competing Interests

The authors declare no competing interests.

## Supplemental Digital Content

Supplemental methods, figures, and tables, https://links.lww.com/ALN/E153

Supplemental methods

Figure S1. Study flowchart

Table S1. Characteristics of the study population

Table S2. Ventilatory and ECMO settings, blood gas analysis, and shunt fraction

## Supplementary Material
