# Supplementary material for: Hemodynamic Effect of Volatile Sedation in Acute Respiratory Distress Syndrome Patients Undergoing Venovenous Extracorporeal Membrane Oxygenation: A Pilot Observational Study
Source: Anesthesiology. 2025 Oct 14;143(5):1411–4. doi: 10.1097/ALN.0000000000005680 (PMC12513033; doi:10.1097/ALN.0000000000005680)
Supplement: Supplementary file 1 [file aln-143-1411-s001.pdf]

Supplementary Material

Hemodynamic Effect of Volatile Sedation in ARDS patients undergoing venovenous ECMO: a pilot observational study.

Marco Giani, Benedetta Fumagalli, Elisa Zoe Battistelli, Marta Frazzei, Giacomo Grasselli, Matteo Pozzi, Emanuele Rezoagli and Giuseppe Foti

Supplementary Methods

VOLATILE SEDATION MANAGAMENT

AnaConDa® is a modified heat and moisture exchanger (HME) specifically designed for the delivery of volatile anesthetics in mechanically ventilated patients. Liquid isoflurane is infused via a syringe pump through a dedicated line into the vaporizer chamber of the device, where a porous rod facilitates the transition from liquid to vapor phase. The resulting anesthetic gas is then carried to the patient with the inspiratory flow generated by the ventilator. AnaConDa is conventionally positioned at the Y-piece, between the breathing circuit and the endotracheal tube. However, in our setting, the AnaConDa was placed on the inspiratory limb of the breathing circuit. This configuration significantly reduces the added dead space - approximately 50-100 mL - which is particularly advantageous in patients receiving ultraprotective ventilation with very low tidal volumes. Furthermore, this setup allows for the use of active humidification, improving overall airway management. Volatile agent concentration is monitored via a side-stream gas analyzer connected to the circuit with a side port, ensuring real-time assessment of end-tidal anesthetic levels. Gas scavenging is achieved through a standard evacuation

system connected to the expiratory outlet of the ventilator, thereby minimizing environmental contamination [4].

At our institution, volatile agents are routinely used as an alternative to intravenous sedatives in patients undergoing controlled mechanical ventilation while on V-V ECMO. Inhaled sedation is maintained until the patient's clinical condition allows for a transition to assisted ventilation, when intravenous sedation is reintroduced.

DATA COLLECTION

Epidemiological data and severity scores were collected at baseline. Hemodynamics, sedative and vasoactive drug dosages, and sedation level were recorded every 2 hours. Ventilator and blood gas parameters were collected every 8 hours; laboratory tests were performed daily.

Supplementary Results

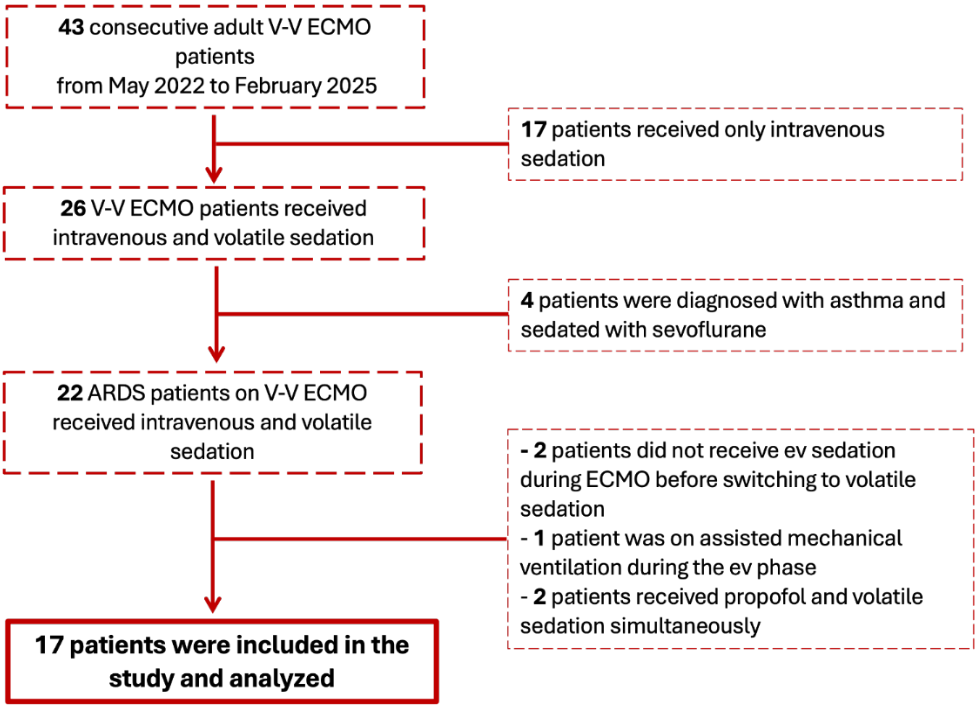

Figure S1. Study Flowchart. Abbreviations: V-V ECMO, venovenous Extracorporeal Membrane Oxygenation; ARDS, Acute Respiratory Distress Syndrome.

**Age**, years	51 [47-57]
**Females**, n (%)	3 (18%)
**Body Mass Index**, Kg/m^2	27 [25-31]
**Etiology**	
Bacterial neumonia, n. (%)	8 (47%)
Viral pneumonia, n. (%)	6 (35%)
Autoimmune disease, n. (%)	2 (12%)
Trauma, n. (%)	1 (6%)

Table S1. Characteristics of the study population.

The median duration of intravenous sedation prior to transitioning to volatile sedation was 3 [IQR 1–5] days, while the median duration of isoflurane sedation was 5 [IQR 3–9]

days. One patient received volatile sedation for less than 24 hours (18 hours) due to clinical improvement and subsequent transition to pressure support ventilation. Hospital mortality was 29%.

Parameter	IV sedation	Isoflurane
**Tidal Volume**, mL/kg IBW	4.4 [3.3-5.3]	4.6 [3.5-5.3]
**Respiratory Rate**, bpm	10 [10-10]	10 [10-10]
**PaO_2**, mmHg	85 [73-95]	86 [77-99]
**$PaCO_2$**, mmHg	47 [44-56]	49 [45-57]
**pH**	7.40 [7.37-7.43]	7.41 [7.36-7.44]
**ECMO blood flow**, L/min	3.3 [2.9-3.4]	3.3. [3.0-3.5]
**ECMO sweep gas**, L/min	4 [3.5-4.5]	4 [3.5-5]
**Pulmonary shunt fraction**, %	57 [51-69]	52 [44-60]

Table S2. Ventilatory and ECMO settings, blood gas analysis and shunt fraction. Abbreviations: IBW, ideal body weight; PaO_2, arterial oxygen tension; $PaCO_2$, arterial carbon dioxide tension; bpm, breaths per minute.
